# Genome-based analysis of Carbapenemase-producing *Klebsiella pneumoniae* isolates from German hospital patients, 2008-2014

**DOI:** 10.1186/s13756-018-0352-y

**Published:** 2018-05-02

**Authors:** Laura Becker, Martin Kaase, Yvonne Pfeifer, Stephan Fuchs, Annicka Reuss, Anja von Laer, Muna Abu Sin, Miriam Korte-Berwanger, Sören Gatermann, Guido Werner

**Affiliations:** 1 Robert Koch Institute, Berlin, Germany; 2 National Reference Centre for Multidrug-resistant Gram-negative Bacteria, Department for Medical Microbiology, Ruhr-University Bochum, Bochum, Germany; 3 Present address: Department of Infection Control and Infectious Diseases, University Medical Centre Goettingen, Goettingen, Germany; 4 Fachgruppe Infektiologie und Hygiene, Landeszentrum Gesundheit North-Rhine Westphalia, Gesundheitscampus 10, Bochum, Germany

**Keywords:** KPC, OXA-48, ST258, Hypermucoviscous, Hypervirulent, ST23, K1 capsule

## Abstract

**Background:**

By using whole genome sequence data we aimed at describing a population snapshot of carbapenemase-producing *K. pneumoniae* isolated from hospitalized patients in Germany between 2008 and 2014.

**Methods:**

We selected a representative subset of 107 carbapenemase-producing *K. pneumoniae* clinical isolates possessing the four most prevalent carbapenemase types in Germany (KPC-2, KPC-3, OXA-48, NDM-1). Isolates were processed via illumina NGS. Data were analysed using different SNP-based mapping and de-novo assembly approaches. Relevant information was extracted from NGS data (antibiotic resistance determinants, *wzi* gene/*cps* type, virulence genes). NGS data from the present study were also compared with 238 genome data from two previous international studies on *K. pneumoniae.*

**Results:**

NGS-based analyses revealed a preferred prevalence of KPC-2-producing ST258 and KPC-3-producing ST512 isolates. OXA-48, being the most prevalent carbapenemase type in Germany, was associated with various *K. pneumoniae* strain types; most of them possessing IncL/M plasmid replicons suggesting a preferred dissemination of *bla*_OXA-48_ via this well-known plasmid type. Clusters ST15, ST147, ST258, and ST512 demonstrated an intermingled subset structure consisting of German and other European *K. pneumoniae* isolates. ST23 being the most frequent MLST type in Asia was found only once in Germany. This latter isolate contained an almost complete set of virulence genes and a K1 capsule suggesting occurrence of a hypervirulent ST23 strain producing OXA-48 in Germany.

**Conclusions:**

Our study results suggest prevalence of “classical” *K. pneumonaie* strain types associated with widely distributed carbapenemase genes such as ST258/KPC-2 or ST512/KPC-3 also in Germany. The finding of a supposed hypervirulent and OXA-48-producing ST23 *K. pneumoniae* isolates outside Asia is highly worrisome and requires intense molecular surveillance.

**Electronic supplementary material:**

The online version of this article (10.1186/s13756-018-0352-y) contains supplementary material, which is available to authorized users.

## Background

Third-generation cephalosporin-resistant and carbapenem-resistant *Klebsiella pneumoniae* are important health-care pathogens associated with increased morbidity and mortality among high-risk patients. Recent data from the European Antimicrobial Resistance Surveillance network (EARS-Net) for health-care associated pathogens (from invasive infections) constitute a rising number of third-generation cephalosporin-resistant and carbapenem-resistant *K. pneumoniae* all over Europe [1]. In the period 2012 to 2015 some countries, such as Spain, Portugal and Croatia, experienced rising trends of carbapenem resistance still at a low level (< 4%) but triggering the overall European trend. In other countries such as Romania, Italy, and Greece, the rates were already much higher and, in addition, partly showed an increasing trend (Romania). In contrast to this, the majority of European countries, including Germany demonstrated rates below 1% without showing any significant development in recent years. Thus, Germany could be considered as a low-prevalence country according to EARS-Net data regarding the occurrence and spread of carbapenem-resistant *K. pneumoniae.* However, the National Reference Centre (NRC) for Gram-negative nosocomial pathogens in Germany is challenged by analysing an increasing number of several thousand carbapenem-resistant *Enterobacteriaceae*, *Pseudomonas aeruginosa* and *Acinetobacter* each year, starting with only a few hundred isolates back in 2008/2009. In 2015 and 2016 diagnostic laboratories throughout Germany sent 1148 and 1317 carbapenem-resistant *K. pneumoniae* isolates to the NRC, respectively, and exactly 51% of them possessed a carbapenemase [2, 3]. The most frequent carbapenemase type in these isolates was OXA-48, followed by KPC-2/− 3 and NDM-1.

Since May 2016, *Acinetobacter* spp. and *Enterobacteriaceae* with carbapenem non-susceptibility are mandatorily notifiable to the national health authorities in Germany. From September 2016 to August 2017, 4028 notifications were reported. The most prevalent notified pathogen was *K. pneumoniae* (1147, 29%). Notifications of carbapenem-resistant *K. pneumoniae* came from all over Germany with a range of 3 to 267 notifications per federal state. For 293 (26%) of those, a carbapenemase was reported. The most common specified carbapenemases were OXA-48-like (53%), KPC-2/− 3 (17%) and NDM-1 (12%)(A.R., A.v.L., M.A.S., unpublished data).

With this study we aimed at describing a population snapshot of carbapenemase-producing *K. pneumoniae* isolated from hospitalized patients in Germany. We compiled a representative subset of 107 carbapenemase-producing *K. pneumoniae* collected between 2008 and 2014 from all over Germany and harbouring carbapenemases OXA-48, KPC-2, KPC-3 and NDM-1. We performed whole genome sequencing to (i) infer the phylogenetic relatedness of corresponding isolates including identification of the multi-locus sequence type (MLST); (ii) extract β-lactamase- and virulence gene content; (iii) deduce the capsule type (*wzi* typing); and (iv) reconstruct the plasmid content. Finally, we compared our results with *K. pneumoniae* isolates from clinical and ambulatory settings in Europe and worldwide (carbapenemase-producers were in the minority).

## Methods

### Strain collection

All carbapenemase-producing *K. pneumoniae* isolates included in the study were collected during 2008 to 2014 by the NRC and the Robert Koch Institute (RKI). We selected a comprehensive subset of 107 strains including only carbapenemase-producing *K. pneumoniae* possessing the four most prevalent carbapenemase types in Germany (KPC-2, KPC-3, OXA-48, NDM-1). Isolates were selected based on a collection of 2104 strains at both participating reference institutions (NRC *n* = 1904; RKI *n* = 200) [2]. The selected sample set also contained type strains representing prominent outbreaks (one isolate per setting) [4–7] and other isolates representing previously unpublished outbreaks which were recognized as part of the routine work of the NRC and RKI. The isolate collection covered 12 of the 16 federal states in Germany (Additional file 1: Figure S1 and Additional file 2: Table S1). The number of carbapenemase-producing *K. pneumoniae* isolates sent to the NRC and RKI increased continuously until 2014; however, we tried to balance the selection of isolates over the collection period appropriately (2010, *n* = 20; 2011, *n* = 20; 2012, *n* = 25; 2013, *n* = 22; 2014, *n* = 13). The distribution of carbapenemase genes within our sample set represents the distribution among the entire set of 2104 clinical carbapenemase-producing *K. pneumoniae* isolates in both reference institutions during the time period analysed (*bla*_*OXA-48*_ *n* = 42, 39%; *bla*_*KPC-2*_ *n* = 34; 32%; *bla*_*KPC-3*_ *n* = 26, 24%; *bla*_*NDM-1*_ *n* = 5, 5%). Although strain sending to the NRC or the RKI is voluntary, the annual collection is comprehensive enough to deduce a broader picture and allow some general assumptions [2, 8].

### Whole genome sequencing (WGS)

Bacteria were grown in Brain Heart Infusion (BHI) broth. DNA was extracted from overnight cultures using the MagAttract Kit (Qiagen, Hilden, Germany) and the DNeasy Blood & Tissue Kit (Qiagen) in line with the manufacturer’s instructions. Qubit dsDNA HS Assay Kit (Invitrogen/Thermo Fisher Scientific, Karlsruhe, Germany) was used for DNA quantification. Sequencing libraries were prepared applying the Nextera XT Kit (Illumina, San Diego, USA) and sequenced on an Illumina Miseq using v3 chemistry (2 × 300 bp) according to the manufacturer’s protocol.

### Mapping-based WGS data analysis

A base quality-dependent trimming was performed for Illumina raw reads applying Trimmomatic [9](version 0.32, parameters: illuminaclip off, slidingwindow 4:15, leading 3, trailing 3, crop off, headcrop off, minlen 36, avgqual off) using BWA-SW (version 0.7.13-r1126, default parameters) [10]. Isolates generating a high number of ambiguous sites (cutoff: ≥15% “N” in the consensus sequence) were excluded from the mapping and tree calculation (see also Results chapter). The reference genome was selected using *refRank* (version 1.0.0; see below). Single nucleotide polymorphisms (SNPs) were called using Varscan v2.3 [11] and variant positions were filtered using *SNPfilter* (version 2.2.0; see below). Based on the aligned variant positions maximum-likelihood trees were calculated using RAxML (version 8.2.7, model GTR GAMMA, 100 bootstraps) [12]. Artificial Illumina reads were generated from the FASTA-sequence downloaded from NCBI using ART (version 2.5.8, parameters: system MiSeq v3, read length 250, paired end, fragment length 600, standard deviation 300, coverage 100) [13] and mapped to the reference genome as described above for the sequenced isolates.

### Reference genome selection using refRank

To optimize the selection of a reference sequence for next generation sequencing (NGS) read alignment (mapping), we created a Python-based application called *refRank*, which provides a coverage-based reference ranking. For this purpose, datasets of paired-end or unpaired reads are aligned against a collection of defined reference sequences using BWA-SW [10] or BWA-MEM (arXiv:1303.3997v2 [q-bio.GN]). Computational costs can be reduced by using only a fraction of randomly picked (paired-end) reads of each dataset (e.g. 10% of all reads). Per base coverage is then determined using SAMtools [14] and normalized to the reference length and the number of total (mapped and unmapped) reads according to a detached formula 1 described in [15]. Dataset-specific reference ranking is based on the calculated C scores. Additionally, a global reference ranking based on all datasets is provided by calculating the grand average of reference-specific C scores. The source code for *refRank* is freely available under the terms of the GNU (https://www.gnu.org/) General Public License v3.0 from https://gitlab.com/s.fuchs/refRank.

In the present study, *refRank* (version 1.0.0) was used with default parameters. The entire collection of completed genomic sequences of *K. pneumoniae* (*n* = 79) available on RefSeq (accessed on 1st Dec 2016) was used as reference dataset. All 105 raw read datasets were trimmed using Trimmomatic (see above) [9, 16]. According to the reference ranking (not shown), the genomic sequence of *K. pneumoniae* KPNIH30 (NZ_CP009872.1) has been selected as a reference for further analysis.

### Variant site filtering using SNPfilter

A python-based application called *SNPfilter* was developed to condense the alignment of all reconstructed sequences to variant positions only and, thus, to significantly reduce computational costs of subsequent phylogenetic analyses. Optionally, sites can be excluded based on (i.) ambiguous base calls and/or deletions; (ii.) SNP accumulation (based on exclusion distance); and (iii.) user-defined regions (based on genomic coordinates). Importantly, circular replicon topologies can be considered when applying an exclusion distance for accumulated variant positions. The output of *SNPfilter* provides different files containing (i.) aligned variant sites that meet the filter criteria (FASTA format); (ii.) general information such as sequence names, number of sites containing ambiguous base calls or gaps, number of variants before and after filtering (TXT format); and (iii.) coordinate-specific filter status and sequence information (CSV format). The source code for *snpFilter* is freely available under the terms of the GNU General Public License v3.0 from https://gitlab.com/s.fuchs/snpFilter [15].

In the present study sites containing gaps or ambiguous base calls were excluded. To exclude SNPs in repetitive regions and phage sequences, the reference sequence *K. pneumoniae* KPNIH30 (NZ_CP009872.1) was analysed using the repeat analysis tool in Kodon (Applied Maths) version 3.62 and the phage search tool PHASTER [16]. Additionally, SNPs in regions of annotated phages and transposases were rejected (not shown). Trees were illustrated using FigTree v1.4.3. (http://tree.bio.ed.ac.uk/software/figtree/; last access: 25/04/2018) and iTOL v3 (https://itol.embl.de/; last access: 25/04/2018).

### De novo assembly

Raw reads were trimmed applying Trimmomatic 16 (version 0.32, parameters: Illuminaclip off, maxinfo 50:0.8, leading 3, trailing 3, crop off, headcrop off, minlen 36, avgqual off). Trimmed reads were de novo assembled using A5-miseq (version 20,150,522) [17].

### WGS-based deduction of MLST, wzi type, β-lactamase and virulence genes

WGS data of all isolates were analysed using Resfinder [18], PlasmidFinder and the MLST tool [19] provided by the Centre for Genomic Epidemiology (https://cge.cbs.dtu.dk/services/; last access: 25/04/2018). Determination of capsular type for *Klebsiella* strains was conducted by *wzi* gene sequencing extracted from contigs derived from de novo assembly [20] and using the websites of the Pasteur Institute (http://bigsdb.pasteur.fr/perl/bigsdb/bigsdb.pl?db=pubmlst_klebsiella_seqdef_public&page=downloadAlleles&scheme_id=4&render=1; last access 25/04/2018). Furthermore, all sequenced genomes were screened for the presence of the following virulence genes or gene clusters using the reference databank of the Pasteur Institute: *mrk*, yersiniabactin, allantionase, colibactin, salmochelin, aerobactin, *kfu*, microcin, *rmpA*, *rmpA2*, and *kvg*. Genes with mismatches or incomplete sequences were additionally analysed in Geneious searching for Stopp codons and inserted IS elements (Geneious v10, Biomatters Lmtd., New Zealand). Primers used for additional and confirmatory PCR amplification of different virulence and β-lactamase genes are given as a Additional file 3: Table S2.

### Phenotypic test for hypervirulence

In order to test for a putative hypervirulence or hypermucoviscous phenotype, we established the so-called ‘string test’ [21]. The hypervirulent *K. pneumoniae* isolate of ST2398 possessing a K2 capsule was used as a positive control [22].

## Results

### MLST analyses

**Fig. 1:**
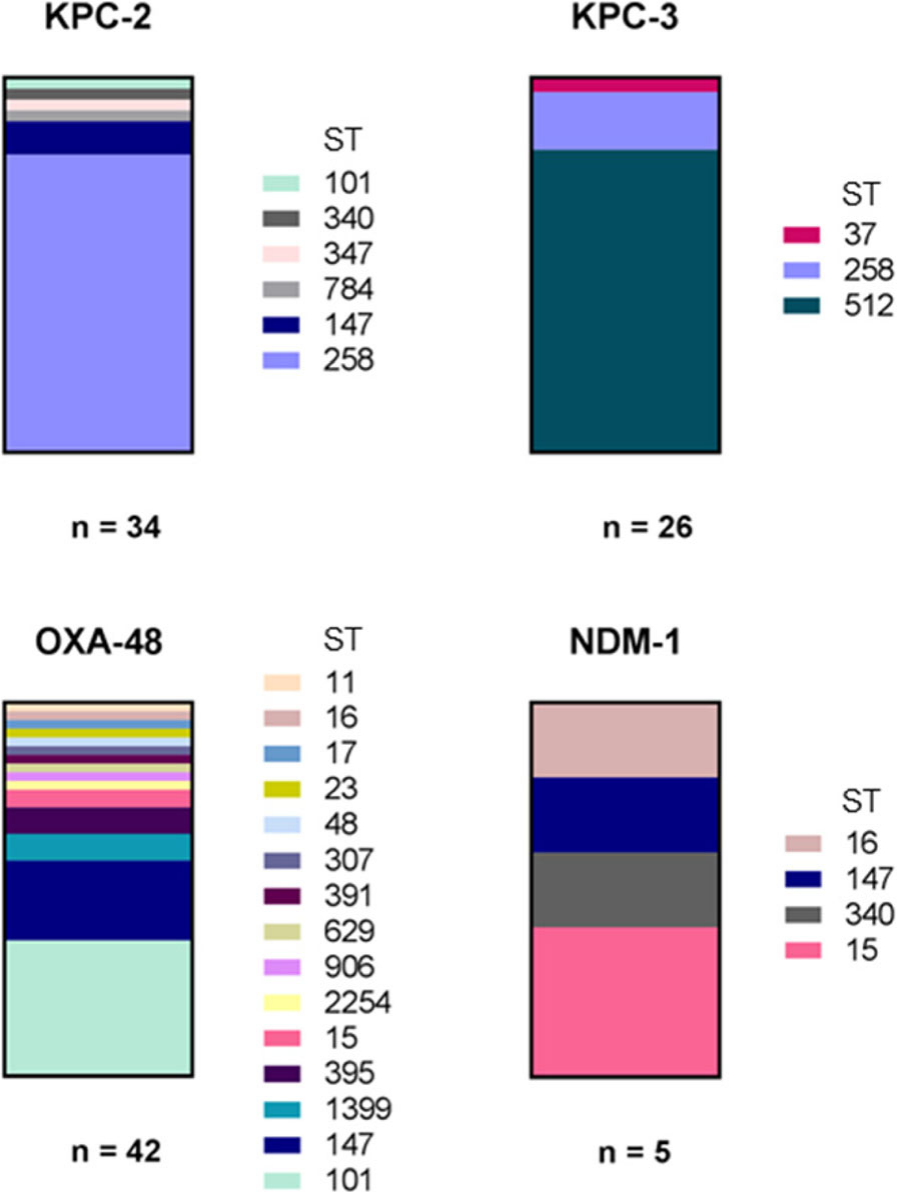
Distribution of *Klebsiella*-MLST and carbapenemase types. The columns show the relative amount of corresponding sequence type (ST) within the group of carbapenemase-producing isolates (KPC-2, KPC-3, OXA-48, NDM-1)

We determined the *K. pneumoniae* MLST types (STs) and the carbapenemase genes from the WGS data. The 107 sequenced isolates belonged to 21 different STs. Figure 1 shows the distribution of STs among the four analysed carbapenemase types (OXA-48, KPC-2, KPC-3, NDM-1). The majority of KPC-2-producing isolates belonged to ST258 (*n* = 27, 80%). Three isolates were ST147 and one each belonged to ST101, ST340, ST347 and ST784. KPC-3-producing isolates demonstrated a low diversity; 21 isolates (81%) were ST512, four were ST258 and one isolate was ST37. Altogether 42 OXA-48-producing isolates revealed 15 different STs, whereas 15 isolates (36%) were ST101 and nine (21%) were ST147. The other STs appeared less than four times. Furthermore, one new ST (ST2254; allelic profile: *gapA* _25, *infB*_33, *mdh*_141, *pgi*_26, *phoE*_7, *rpoB*_1, *tonB*_56) with the carbapenemase gene *bla*_OXA-48_ was identified. The five NDM-1-producers that were included in this study could be assigned to four different STs (ST15, ST16, ST147 and ST340).

### Content of additional β-lactamase genes

Besides the four corresponding carbapenemase genes, a number of additional β-lactamase genes were determined from WGS data using ResFinder (Table 1). Five variants of the chromosomally encoded SHV β-lactamase were identified. SHV-11 was the most frequent subtype found in 68% of isolates. However, one OXA-48-producing and one KPC-2-producing isolate did not show any *bla*_SHV_ gene but a *bla*_LEN_ gene indicative of the species *Klebsiella variicola*. Furthermore, the majority of isolates possessed *bla*_TEM-1_ (80%); 28% contained *bla*_OXA-1_ and 11% *bla*_OXA-9_. Additional extended-spectrum β-lactamase (ESBL) genes *bla*_CTX-M-15_ and *bla*_CTX-M-14_ were found in 24% and 4% of the isolates, respectively. OXA-48- and NDM-1-producing isolates possessed an additional ESBL gene more often than KPC-2- and KPC-3-producing isolates (Table 1).

**Table 1:** Additional β-lactamase genes in carbapenemase-producing *K. pneumoniae* isolates. Counts are given in exact numbers and in percentages. Genes were extracted from NGS data using ResFinder software

	KPC-2	KPC-3	OXA-48	NDM-1	Total
	*n* = 34	*n* = 26	*n* = 42	*n* = 5	*n* = 107
Chromosomal β-lactamase genes					
*bla*_SHV-1_	2 (6%)	–	16 (38%)	1 (20%)	19 (18%)
*bla*_SHV-11_	25 (74%)	25 (96%)	21 (50%)	2 (40%)	73 (68%)
*bla*_SHV-12_	6 (18%)	–	–	–	6 (6%)
*bla*_SHV-28_	–	–	3 (7%)	2 (40%)	5 (5%)
*bla*_SHV-77_	–	–	1 (2%)	–	1 (1%)
*bla*_LEN-16_	–	–	1 (2%)	–	1 (1%)
*bla*_LEN-27_	1 (3%)	–	–	–	1 (1%)
Acquired β-lactamase genes					
*bla*_TEM-1_	25 (74%)	23 (88%)	34 (81%)	3 (60%)	85 (80%)
*bla*_OXA-1_	–	1 (4%)	27 (64%)	2 (40%)	30 (28%)
*bla*_OXA-9_	1 (3%)	–	10 (24%)	1 (20%)	12 (11%)
*bla*_CTX-M-14_	–	–	4 (10%)	–	4 (4%)
*bla*_CTX-M-15_	1 (3%)	1 (4%)	15 (36%)	5 (100%)	22 (21%)
*bla*_CMY-2_	–	–	1 (2%)	–	1 (1%)
*bla*_VEB-1_	1 (3%)	–	–	–	1 (1%)

### Content of virulence genes

All but one of the 107 investigated isolates contained three or less of the tested virulence genes or gene clusters. The most frequent gene cluster was *mrk* encoding regulation and expression of the type 3 pilus. Genes *mrkA*, *mrkB*, *mrkC*, *mrkD*, *mrkF*, *mrkH*, *mrkI* and *mrkJ* were identified in all isolates. However, the *mrkH* gene was interrupted by an insertion of an IS element in 29 isolates; one of which additionally showed insertion of an IS element in *mrkI*. Another isolate showed an insertion within the *mrkJ* gene. Correspondingly, only 77 isolates (72%) possessed an entire *mrk g*ene cluster (Additional file 1: Figure S2). Strikingly, in 26 of 29 strains the insertion site in *mrkH* was completely identical. The ORF was destroyed after 207 nucleotides by the introduction of an IS element (93% identity to IS*Ec21* according to ISfinder [23]). These 26 isolates were KPC-2 producers of ST258 (*wzi*_29). Yersiniabactin was the second most frequent virulence factor. Two isolates possessed a premature STOP codon in each gene (*ybtA* and *irp2*). Accordingly, 36% of all isolates harboured a complete and putatively functional yersiniabactin cluster. The *kfu* gene cluster was identified in 21% (*n* = 22) including all isolates of ST101 (*n* = 16) and ST15 (*n* = 4). Genes encoding the siderophore aerobactin were detected in six isolates.

The single ST23 isolate producing OXA-48 (no. 316/15) was exceptional by harbouring a great repertoire of virulence genes showing hits for all tested targets except *kvg* (Additional file 3: Table S3). The ORF of *rmpA2* demonstrated a premature stop codon (see also Discussion). Due to the high number of virulence genes the so-called ‘string test’ was performed to identify a putative hypermucoviscous phenotype associated with hypervirulence; however, the test result was repeatedly negative.

### Identifying the capsule type (*wzi* typing)

NGS data were used to extract *wzi* allele variants which finally deduced the corresponding capsule type [20]. Details are given in Additional file 3: Table S4. Altogether 25 *wzi* alleles were identified including five new variants that received new allele types after submission to the corresponding database at the Pasteur Institute, Paris. One isolate did not reveal any *wzi* result, either from analysing NGS data or from performing *wzi*-specific PCRs (Additional file 3: Tables S2/S4; isolate 274/15, ST147). Isolates of a single ST did not always possess the same *wzi* allele. For instance, *K. pneumoniae* ST15 isolates possessed three *wzi* alleles (*wzi*_24, *wzi*_19 and *wzi*_new1). Isolates of ST147 also possessed three different allele types; however, the vast majority were associated with *wzi*_64. Eighty-four percent (26/31) of ST258 isolates contained the *wzi*_29 allele, which is equivalent to capsule cluster type *cps*1. All these isolates also produced KPC-2. In contrast, all KPC-3-producing ST258 isolates showed *wzi*_154 (associated with the *cps2* cluster). All isolates of ST512 (*n* = 21) contained *wzi*_154. The single ST23 isolate no. 316/15 possessing a high number of virulence genes possessed *wzi*_1 which is associated with capsule type K1. It has been described that *K. pneumoniae* with capsule type K1 belong to the group of hypervirulent strain types [24].

### Plasmid content

**Fig. 2:**
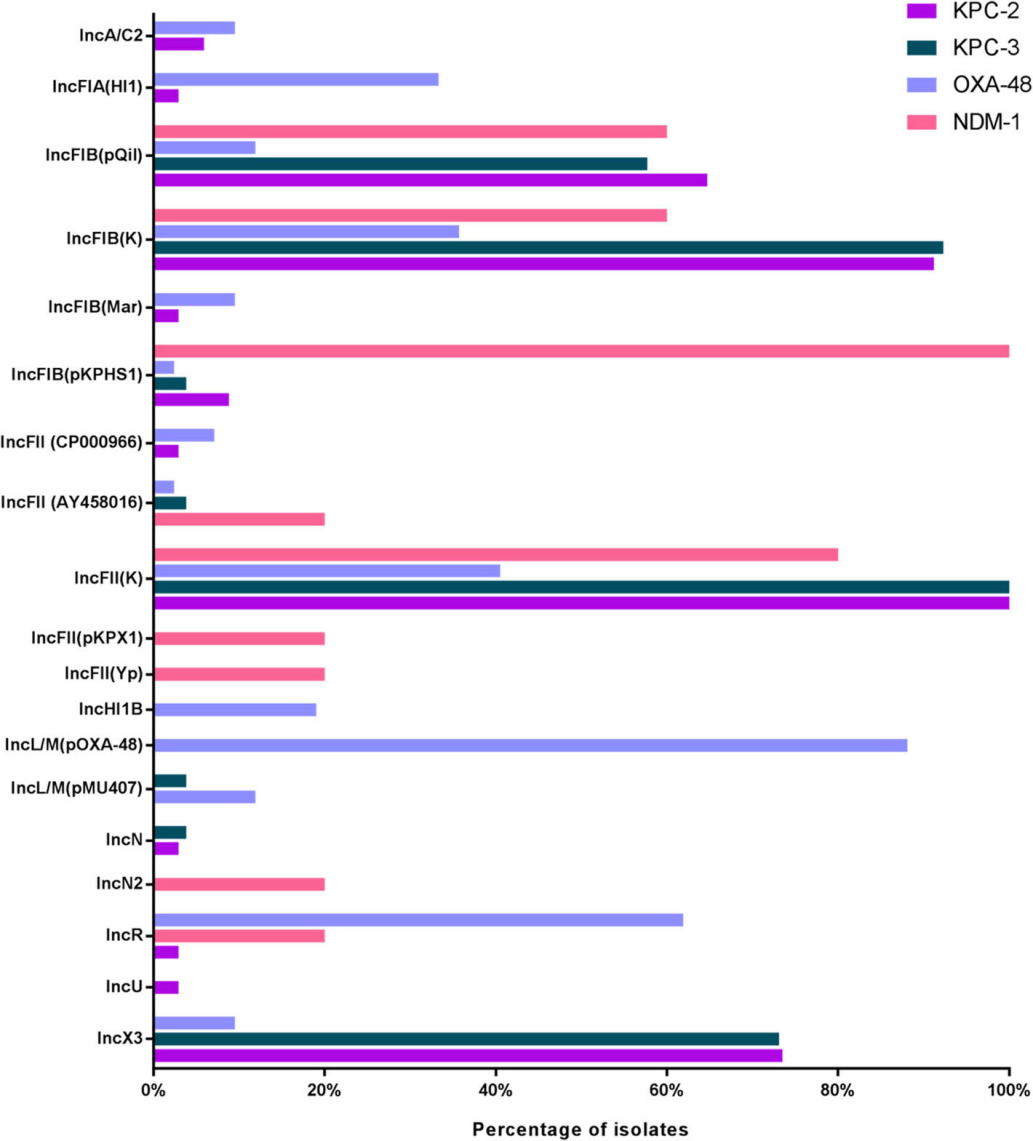
Identified plasmid replicons in 107 carbapenemase-producing *K. pneumoniae* isolates using NGS data and PlasmidFinder software. The inc groups were presented at the y axis and the color code refers to the corresponding carbapenemase genes (see legend)

Using the tool PlasmidFinder the plasmid content and the equivalent incompatibility (Inc) groups were extracted from NGS data (Fig. 2). Most frequent were IncFII(K) and IncFIB(K) occurring in 76% and 68% of all isolates, respectively. All KPC-2- and KPC-3-producing isolates possessed IncFII(K) and approximately 90% also possessed IncFIB(K). Seventy-three percent of KPC-2- and KPC-3-producing isolates were positive for IncX3. All NDM-1-producing isolates showed a positive result for IncFIB whereas all OXA-48-producing *K. pneumoniae* isolates possessed IncL/M (IncL/M(pMU407) or IncL/M(pOXA-48)). Furthermore, the majority of OXA-48-producing isolates contained an IncR plasmid (62%).

### Phylogeny of carbapenemase-producing *K. pneumoniae* isolates from Germany

Searching for β-lactamase genes in NGS data revealed two isolates with a *bla*_LEN_ gene indicative of the species *K. variicola* (isolates no. 275/15 and no. 328/15). In silico restriction of *gyrA* with *Taq*I und *Hae*III confirmed their association with phylogroup III and species *K. variicola*, respectively. Accordingly, these two isolates were not considered for subsequent phylogenetic analysis.

**Fig. 3:**
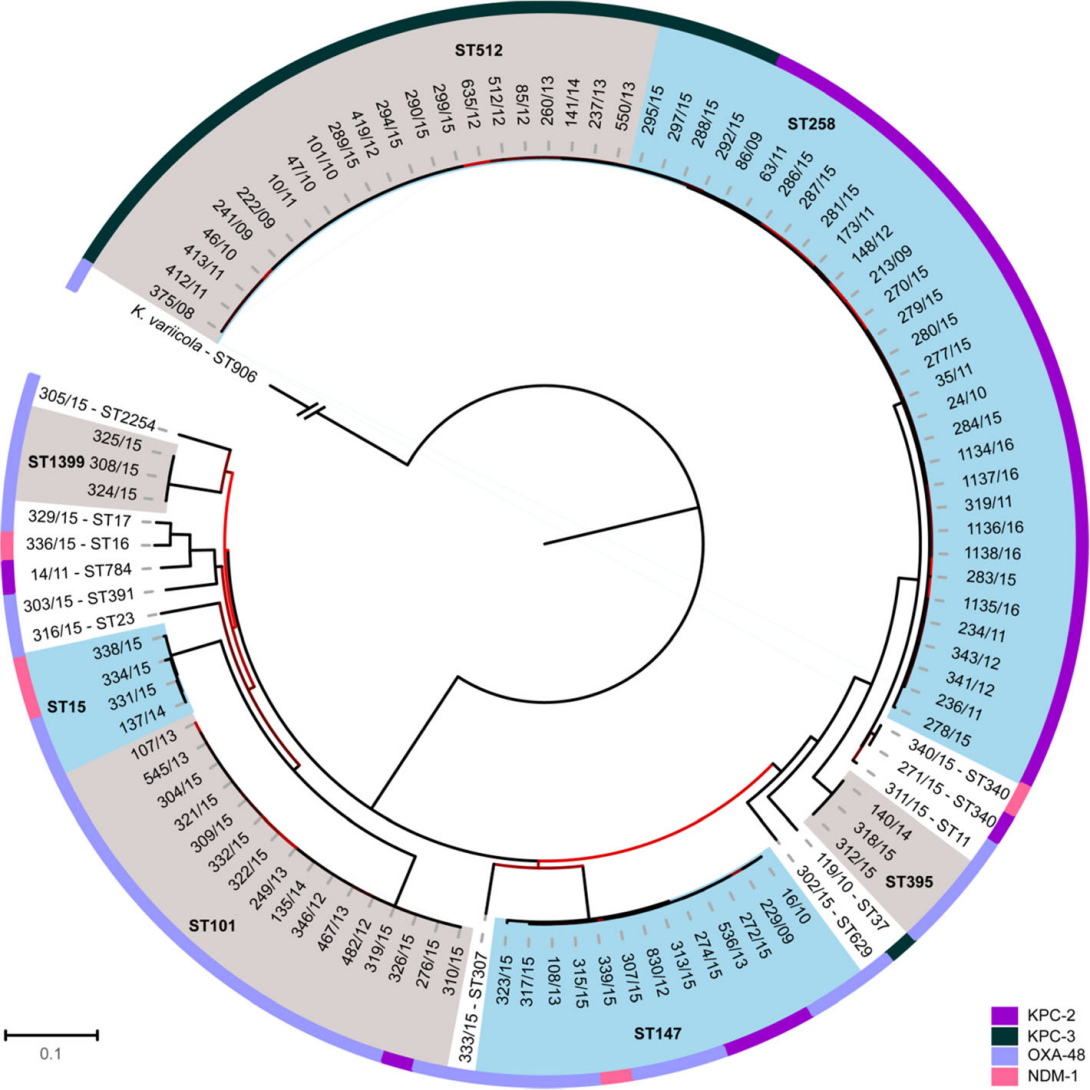
Phylogeny of 103 carbapenemase-producing *K. pneumoniae* isolates from German hospital patients, 2008-2014. The image shows a ML tree based on 159.154 SNPs of the core genome. A *K. variicola* isolate (ST906, OXA-48) served as an outgroup to root the tree (the corresponding branch is shortened 1/100). Clades of three or more isolates received an alternating colour code grey/blue. The clades are labelled with the corresponding MLST type. The outer ring corresponds to the four different carbapenemase (see Figure legend for colour code). The colour of the branches correlate with the corresponding boostrap value (light red (0) to black (100))

Phylogenetic comparison was done using a reference-based mapping approach. The best reference was selected by *refRank*, suggesting *K. pneumoniae* isolate *KPNIH30* (NCBI-no. NZ_CP009872.1) as best match. One of the *K. variicola* isolates (ST906, OXA-48) was included to root the tree. Two *K. pneumoniae* isolates (no. 301/15 [*bla*_*OXA-48*_; ST16] and no. 330/15 [*bla*_*OXA-48*_; ST48]) were excluded from the mapping and tree calculation due to a high number of ambiguous sites (cutoff: ≥15% “N” in the consensus sequence). After excluding repetitive regions and mobile genetic elements, 159,154 SNP positions (length of the reference genome: 5,306,618 bp) remained to construct the maximum likelihood tree (Fig. 3).

Among the 103 analysed carbapenemase-producing *K. pneumoniae* isolates four major clusters could be identified corresponding to sequence types ST258, ST512, ST147, and ST101. A strong association between the MLST type and carbapenemase type was noticeable for isolates of ST258, ST512, and ST101. The ST512 isolates contained KPC-3 exclusively. Furthermore, the vast majority of ST258 isolates possessed KPC-2. All but one isolates of ST101 produced OXA-48. In contrast, ST147 isolates produced mainly OXA-48, and to a lesser extent also KPC-2 and NDM-1. Isolates of ST11 and ST340 constitute a clonal group CG258 together with isolates of ST258 and ST512, a detailed view is given in Additional file 1: Figure S3. ST512 isolates formed a sub-cluster within CG/ST258 isolates. Within the CG/ST258 cluster three subgroups were noticeable which correlate with the results of the *wzi* typing. All isolates of ST258 associated with the *wzi*_29 built a separate cluster as well as the four ST258 isolates with the *wzi*_154 allele. The latter were located on a separate branch and clearly different from ST512 possessing the *wzi*_154 allele as well. One single isolate of ST258 possessed *wzi*_83, but was clearly separated from all other CG258 isolates. The association of the *wzi* allele and the carbapenemase type was even stricter than with the sequence type; *wzi*_154 was always linked to KPC-3 and *wzi*_29 was exclusively associated with KPC-2.

### Phylogeny of carbapenemase-producing *K. pneumoniae* isolates from Germany in the international context

**Fig. 4:**
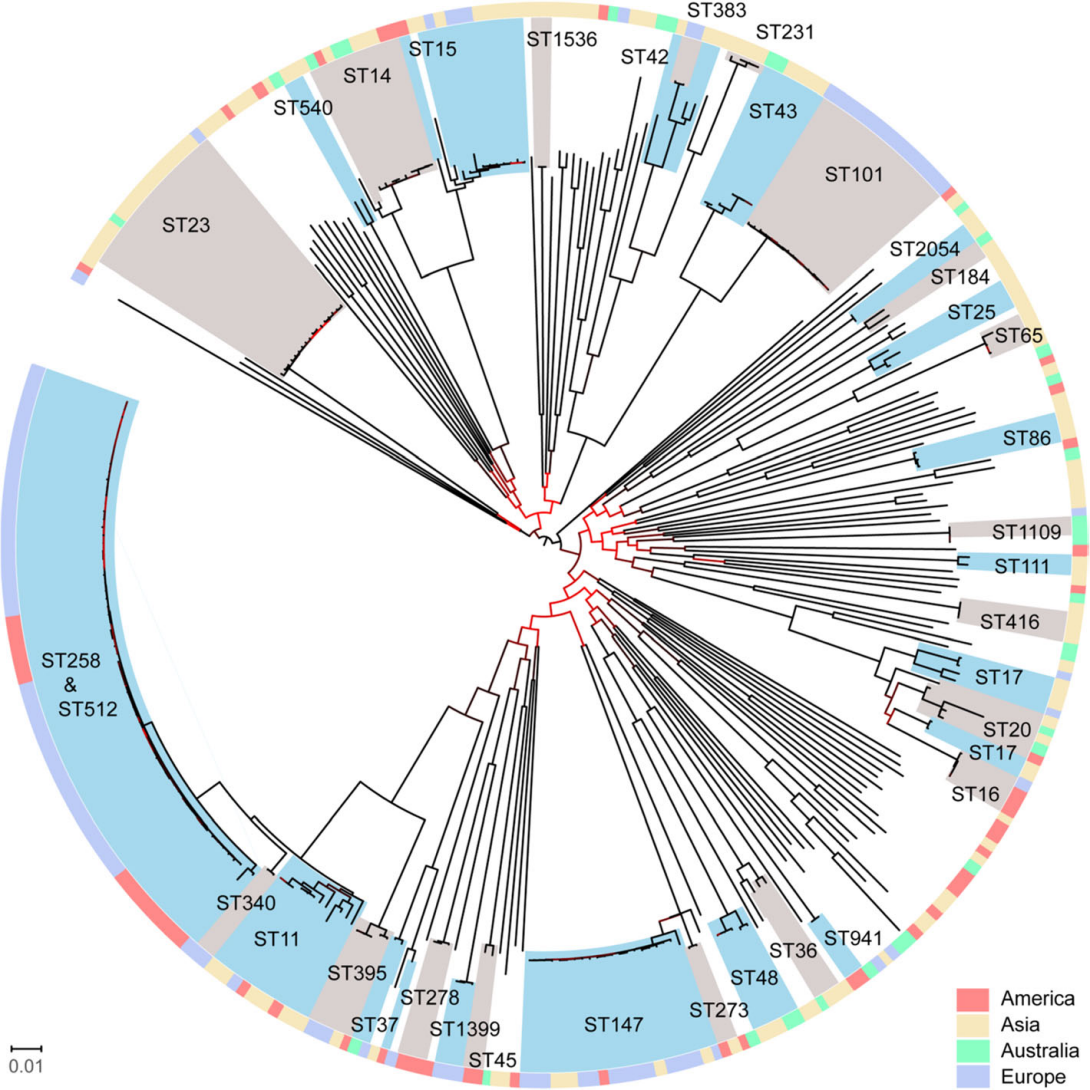
Phylogeny of carbapenemase-producing isolates from Germany, 2008-2014 in relation to an international *K. pneumoniae* isolate collection. The image shows a ML tree based on 126.347 SNPs for 99 carbapenemase-producing isolates from Germany and 238 international *K. pneumoniae* isolates. The tree was rooted midpoint. Clades represented by two or more isolates were colour-coded/shaded (grey/blue) and the MLST type is listed. The outer ring corresponds to the origin of the strains given as a colour code (see Figure legend). The colours of the branches correlate with the corresponding boostrap value (light red (0) to black (100))

To reconstruct a phylogeny of the German carbapenemase-producing *K. pneumoniae* isolates in relation to clinical *K. pneumoniae* from other countries, we performed NGS-based analysis with *K. pneumoniae* genomes from our study and two other collections (irrespective of the carbapenemase type or resistance phenotype in general): all human *K. pneumoniae* isolates (KpnI) described in the study by Holt et al., 2015 [25] and all completely closed *K. pneumoniae* genomes in the *ncbi* Genome Database (https://www.ncbi.nlm.nih.gov/genome/; 12/2016). In preparation of the analysis, completely closed genomes were artificially separated into MiSeq reads and subsequently all NGS data from MiSeq and HiSeq [25] runs were subjected to a reference-based mapping as described before but using *K. pneumoniae subsp. pneumoniae* NTUH-K2044 (NCBI-no. NC_012731.1; was also used as a reference by Holt et al., [25]). Isolates with more than 15% ambiguities were excluded from subsequent analyses resulting in a tree consisting of 337 *K. pneumoniae* isolates including 99 isolates from our study and 238 isolates from the other two studies (Holt collection: 167; ncbi: 71). After excluding repetitive regions and mobile genetic elements 126,347 SNPs remained to reconstruct an ML tree (Fig. 4). The majority of the other isolates originated from Asia (*n* = 139), followed by America (*n* = 59) and Australia (*n* = 33). Furthermore, the collection included seven European isolates, three from Greece; one each from Switzerland and France and two from Germany (independent from the present collection). NGS analyses revealed 116 MLST types within the whole collection, 36 of which appeared more than once (the MLST type of 16 isolated could not be determined). The 238 international isolates comprised 108 different MLST types, 33 of which appeared more than once, demonstrating a much higher diversity than identified for the 99 isolates from this study from Germany only (21 MLST types). The largest clusters were formed by isolates belonging to ST11, ST14, ST15, ST23, ST101, ST147, ST258 and ST512. The ST14 cluster only contained isolates from international collections whereas the ST101 cluster exclusively showed isolates from Germany. Clusters ST15, ST147, ST258, and ST512 demonstrated an intermingled subset structure consisting of German and international *K. pneumoniae* isolates. ST23 was the most frequent MLST type in Asia but was found only once in Germany.

We also took a closer look into the CG/ST258 cluster isolates (66 of the 337 isolates; Additional file 1: Figure S4). The majority of isolates derived from the German collection (*n* = 48), another 18 were deposited in GenBank as complete genomes and originated from America/USA (*n* = 17) and Switzerland (*n* = 1). All international ST258 and ST512 isolates also possessed a carbapenemase gene. All American isolates were of ST258 and the Swiss isolate was ST512. All but two isolates contained the *wzi*_154 or *wzi*_29 alleles (*wzi*_150 in isolate NZ_CP010392.1; *wzi*_83 in no. 278/15). The two isolates demonstrating unique *wzi* alleles were also distantly related to the rest. A strong association between carbapenemase type and *wzi* allele type was identified, as already shown for the German isolates; KPC-2 was always linked to *wzi*_29 and KPC-3 to *wzi*_154. Within the ST258 group, isolates possessing KPC-2 (*wzi*_29) from Germany and America formed two separate sub-clusters. The same situation was noticeable for ST258 possessing KPC-3 (*wzi*_154). Neither finding indicates a recent exchange of isolates between Germany and the US. In contrast, the Swiss isolate (NZ_CP015822.1) clustered together with all the German ST512 isolates.

## Discussion

More than half of the study sample (58/105; 2× *K. variicola*) belonged to the prominent and worldwide distributed clonal group CG258, including ST258, ST512, ST11, ST395, and ST340. In collections from other countries and continents ST258 and ST11 were the most frequent sequence types within CG258 [26]. Isolates of ST258 are worldwide frequently associated with KPC-2, a finding which was also confirmed for the situation in Germany. SNP-based analyses of ST258/KPC-2 and ST512/KPC-3 showed a clear genetic separation of isolates from Europe and the United States (Additional file 1: Figure S4). This indicates a circulation of KPC clones in Europe. The first outbreak due to *K. pneumoniae*-ST258 with KPC-2 was reported in 2008 in Germany, and the index patient had been previously hospitalized in Greece [27]. Routine screening for carbapenemase-producing *K. pneumoniae* is mandatory at hospital admission in Germany only as part of a risk-based adapted approach (considering certain risk factors such as having received hospital treatment in a high-risk country) and thus patients with undetected colonization may contribute to their further spread within Germany [8].

In contrast to ST258, carbapenemase-producing ST11 (CG258) isolates are quite frequent in Asia. In our collection we found only one ST11 isolate indicating that this ST has so far occurred only rarely in Germany. ST395 isolates, representing another CG258 type, appeared three times in our collection and were always associated with OXA-48. OXA-48-producing *K. pneumoniae* isolates of ST395 were also found recently in other European countries, such as France, the Netherlands and Hungary [28–32].

The present collection also contained a cluster of ST101 isolates (*n* = 16), 15 of which were OXA-48 producers. Although the two external collections included in the phylogenetic analysis did not contain ST101 *K. pneumoniae*, the combination ST101-OXA-48 is not unique to Germany. Clinical isolates with these characteristics were described in neighbouring European countries in recent years, such as Czech Republic, France and Belgium [28, 33, 34], and also all over Europe and North Africa [29, 35]. ST101 isolates seem to be especially distributed in Europe, and also mainly associated with OXA-48 [36]. Two further clusters were formed by ST147 and ST15 isolates producing mainly OXA-48 but also NDM-1 and/or KPC-2 (Fig. 3). Both clonal lineages of *K. pneumoniae* are well known as colonizers or the cause of infection in human patients; outbreaks due to ST147 with OXA-48 have been reported in Germany and ST15 with OXA-48 was also found in pets in a German veterinary clinic [37, 38].

Due to the presence of the *bla*_LEN_ gene and the *gyrA* sequence two of the 107 isolates were classified as *K. variicola* (phylogroup Kpn III). The same MLST scheme is used for all three phylogroups and all species *K. variicola, K. pneumoniae* and *Klebsiella quasipneumoniae*, respectively [36]. The OXA-48 producing *K. variicola* isolate of ST906 represents a very rare ST and ST/carbapenemase combination. ST906 was first defined in a set of strains from an Israeli clinic [39]. However, the authors failed to proof the species *K. variicola*. *K. variicola* isolates of ST347 were described in previous studies from China, Japan, Greece and Sweden [40–43]. However, only the Greek isolate was a carbapenemase producer (KPC-2) [42]. In general, reports about carbapenemase-producing *K. variicola* are rare; we found a report about OXA-181-producing *K. variicola* from vegetables imported from Asia to Europe [44] and NDM-producing isolates originating from a river in China [45]. In a very recent study performed in the US an ST906-isolate producing NDM-1 was also identified as *K. variicola* [46]*.*

One isolate was clearly separated from the rest of our collection regarding the presence of virulence genes: the OXA-48-producing ST23 isolate no. 316/15, carrying capsule type K1 and containing all but one tested virulence markers including different siderophore genes (Additional file 3: Table S3). ST23 isolates are known to be hypervirulent and can cause severe infections in apparently healthy individuals [47, 48]. In particular, ST23 isolates expressing the K1 capsule were frequently associated with cases of community-acquired pyogenic liver abscesses and occurred predominantly in Asian countries [24, 48]. Our single ST23 isolate originated from a tracheal secretion suggesting a respiratory tract infection. Subsequent molecular analyses by *Xba*I macrorestriction and pulsed field gel electrophoresis of isolates from the same diagnostic laboratory revealed that this isolate belonged to a cluster of seven isolates that occurred in the period February 2012 to July 2012. The sample isolates originated from three blood cultures, one urine sample and several swabs. No further clinical or patient information was available for these isolates. Hypervirulent strains of *K. pneumoniae* often express a typical hypermucoviscous phenotype and demonstrate a small spectrum of resistances, mainly only to penicillins, e.g. ampicillin. Our ST23 isolate contained various β-lactamase genes, including classical β-lactamases (*bla*_SHV-11_, *bla*_TEM-1_, *bla*_OXA-1_), ESBLs (*bla*_CTX-M-15_) and carbapenemases (*bla*_OXA-48_). A quick, so-called ‘string test’ for identifying a hypermucoviscous phenotype was negative, possibly associated with the premature STOP codon in the *rmpA2* gene (regulator of the mucoid phenotype A gene). However, the string test gives only a hint and is not reliable enough to predict a corresponding clinical behaviour. In addition, we do not know whether this mutation was also prevalent in the other clonally related isolates that were not available for a genome-based analysis. The combination of hypervirulence and resistance to last-line therapeutics is highly worrisome and has just recently raised serious public health concerns [49–51].

A number of recent reports have suggested that the *wzi* locus is frequently exchanged between *K. pneumoniae* isolates [52, 53] and, as such, could be putatively genetically linked to other, horizontally exchanged markers, such as plasmid-located carbapenemase genes. In particular, the highly successful ST258 clone seemed to be a hybrid strain with the *cps* locus (containing the *wzi* gene) located next to a recombination hotspot [54]. Although KPC-2/3, OXA-48 and NDM-1 genes are commonly located on plasmids, a chromosomal integration is also reasonable, at least for some carbapenemase genes. It has been described recently, that *bla*_KPC-2_ could also be chromosomally determined via IS-mediated Tn*4401* transposition into specific chromosomal loci, especially observed in CG258 isolates (ST11, ST258, ST340) [55]. Although we could not properly elucidate plasmid structures by assembling Miseq reads, we suggest a plasmid localization of the carbapenemase genes in most cases based on the sum of assembled and interpreted NGS data. The finding of IncFII sequences in KPC-producing CG258 isolates and IncL/M sequences in all OXA-48 producers supports our suggestion because these associations of carbapenease genes and plasmid replicon types are well known [53, 56].

ST258 isolates (our collection and genomes of international isolates included in the study) contained four *wzi* alleles; *wzi*_29 (*n* = 26); *wzi*_154 (*n* = 4); *wzi*_83 (*n* = 1) and *wzi*_150 (*n* = 1). Similar distributions were described in two recent studies performed in the US [52, 57] The association within ST258 isolates of *cps*1/*wzi*_29 with *bla*_KPC-2_ and *cps*2/*wzi*_154 with *bla*_KPC-3_, respectively, was identified for our study sample of German clinical isolates but was also described for isolates from other studies and countries [54, 58, 59]. Bowers et al., supposed that the association of *cps*1 in KPC-2-producing *K. pneumoniae* isolates and of *cps*2 in KPC-3-positive isolates is derived from the fact that the point mutations in *bla*_KPC_ leading to a differentiation into *bla*_KPC-2_ and *bla*_KPC-3_ appeared at the same time when the two clades with different *cps* loci diverged [58]. All of the ST512 isolates in our study contained *bla*_KPC-3_ and *wzi*-154; which is identical to all *bla*_KPC-3_-positive *K. pneumoniae* isolates of ST258 in a recent study performed in Italy. Thus, our results presented for German isolates confirm previous findings performed on *bla*_KPC_-positive CG258 isolates (ST258, ST512) from Italy [59]. In all ST512 isolates from Italy only *wzi*_154 was present. This exclusive association among a comprehensive set of 461 *K. pneumoniae* isolates collected all over the country led the authors of the previous study to assume that ST512 isolates may have originated from ST258-*cps*2-clade strains [59].

### Limitations

Our study has some limitations. First, strain sending to reference laboratories is voluntary and taking this into consideration we cannot exclude any bias in our strain collection, which served as the basis to select representative isolates for further genome-based analysis. Second, clinical and patient data available with the strains submitted were limited. We could not perform any analysis in this regard, nor could we provide additional clinical and patient information to the data that are already provided. Third, we are well aware that even a collection of about 100 strains representing 12 federal states in Germany and a timeframe of 7 years does not allow statistically significant assumptions (which we have not calculated and specified as such). Fourth, it has already been addressed in previous papers that plasmid content is difficult to reconstruct with Miseq data, thus the location of the carbapenemase gene on a specific plasmid could and, in consequence, a potential spread of distinct carbapenemase gene-carrying plasmids not be identified. Correspondingly, the putative link between plasmid types and carbapenemase genes remains to a certain extent hypothetical. Fifth, in the last 2 years, data from the NRC showed a rapid increase of *Enterobacteriaceae* (including *K. pneumoniae*) isolates producing VIM-1 in Germany [2], but this trend could not be considered when this study was initiated.

## Conclusion

Using WGS-based analysis we were able to describe a population snapshot of clinical, carbapenemase-producing *K. pneumoniae* isolates from hospitalized patients in Germany collected between 2008 and 2014. This revealed a preferred prevalence of KPC-2-producing ST258 isolates and KPC-3-producing ST512 isolates. OXA-48 is the most prevalent carbapenemase type in Germany and was shown to be associated with various *K. pneumoniae* strain types, most of them possessing IncL/M plasmid replicons, suggesting a preferred dissemination of *bla*_OXA-48_ via this well-known plasmid type. We identified a supposed hypervirulent ST23 strain of capsule type K1-producing OXA-48, which turned out to be representative for an entire cluster of infections in a single German health-care centre in 2012. This finding demonstrated that this highly pathogenic and multidrug-resistant *K. pneumoniae* strain type, associated with a serious public health threat outside Asia and within Europe, had already appeared several years ago.

## Additional files

Additional file 1: **Figure S1.** Origin of the 107 German carbapenemase-producing *K. pneumoniae* isolates. Regions are shown, where isolates originated from. Isolates from Saxony could not be elucidated further due to the lack of additional geographic information. Number of isolates is given by the size of the circle (see legend). Image is from: © Bundesamt für Kartographie und Geodäsie, Frankfurt am Main, Germany. **Figure S2.** Virulence gene content in 107 carbapenemase-producing *K. pneumonaie* isolates from Germany. Data are given in % of isolates showing possession of the corresponding gene cluster. The graph shows four most frequent virulence genes identified in more than one single isolate. **Figure S3.** Detailed view of the ML tree concerning ST258/ST512 – carbapenemase-producing *K. pneumoniae* isolates from Germany, 2008-2014. The image shows a subtree of Fig. 3 containing 52 isolates of ST258 (light violett) and ST512 (grey). Colour codes of the inner ring designate the corresponding carbapenemase type, the outer designates the *wzi* allele (see legend). **Figure S4.** ML tree of NGS-based analysis of German *K. pneumoniae* isolates and isolates from an international collection - detailed view of the cluster ST258/ST512 isolates. The image shows a subtree of Fig. 4 containing 66 isolates of ST258 and ST512. Colour codes of the inner ring correspond to the origin of strains, the middle ring to the carbapenemase KPC-2 or KPC-3, and the outer ring demonstrates the *wzi *allele type. (PPTX 1367 kb)

Additional file 2: **Table S1.** Metadata of the 107 German carbapenemase-producing, clinical *Klebsiella *isolates, 2008-2014. Isolates were ordered by carbapenemase type. For more information see main text document. (XLSX 54 kb)

Additional file 3: **Table S2.** Names and sequences of used primers for MLST and PCR amplification of virulence genes and β-lactamase genes. **Table S3.** Identified virulence genes in *K. pneumoniae* isolate no. 316/15 (ST23, OXA-48). **Table S4.** Identified *wzi *alleles from NGS data of 107 carbapenemase-producing *K. pneumoniae* from Germany. (DOCX 35 kb)
