# Supplementary material for: Genome-based analysis of Carbapenemase-producing Klebsiella pneumoniae isolates from German hospital patients, 2008-2014
Source: Antimicrob Resist Infect Control. 2018 May 2;7:62. doi: 10.1186/s13756-018-0352-y (PMC5930415; doi:10.1186/s13756-018-0352-y)
Supplement: Supplementary file 1 — Figure S1. Origin of the 107 German carbapenemase-producing K. pneumoniae isolates. Regions are shown, where isolates originated from. Isolates from Saxony could not be elucidated further due to the lack of additional geographic information. Number of isolates is given by the size of the circle (see legend). Image is from: © Bundesamt für Kartographie und Geodäsie, Frankfurt am Main, Germany. Figure S2. Virulence gene content in 107 carbapenemase-producing K. pneumonaie isolates from Germany. Data are given in % of isolates showing possession of the corresponding gene cluster. The graph shows four most frequent virulence genes identified in more than one single isolate. Figure S3. Detailed view of the ML tree concerning ST258/ST512 – carbapenemase-producing K. pneumoniae isolates from Germany, 2008-2014. The image shows a subtree of Fig. 3 containing 52 isolates of ST258 (light violett) and ST512 (grey). Colour codes of the inner ring designate the corresponding carbapenemase type, the outer designates the wzi allele (see legend). Figure S4. ML tree of NGS-based analysis of German K. pneumoniae isolates and isolates from an international collection - detailed view of the cluster ST258/ST512 isolates. The image shows a subtree of Fig. 4 containing 66 isolates of ST258 and ST512. Colour codes of the inner ring correspond to the origin of strains, the middle ring to the carbapenemase KPC-2 or KPC-3, and the outer ring demonstrates the wzi allele type. (PPTX 1367 kb) [file 13756_2018_352_MOESM1_ESM.pptx]

## Slide 1
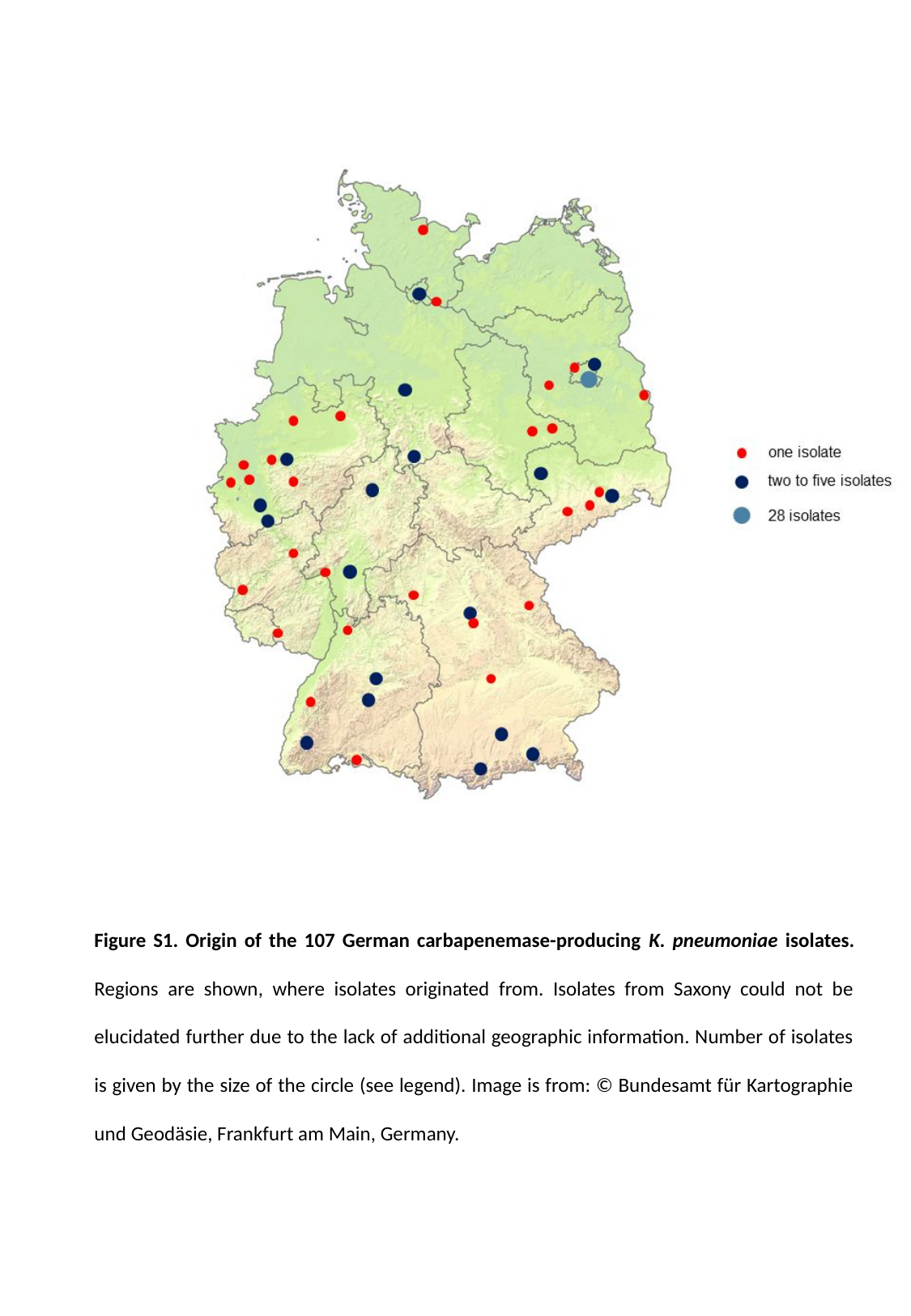

Figure S1. Origin of the 107 German carbapenemase-producing K. pneumoniae isolates. Regions are shown, where isolates originated from. Isolates from Saxony could not be elucidated further due to the lack of additional geographic information. Number of isolates is given by the size of the circle (see legend). Image is from: © Bundesamt für Kartographie und Geodäsie, Frankfurt am Main, Germany.

## Slide 2
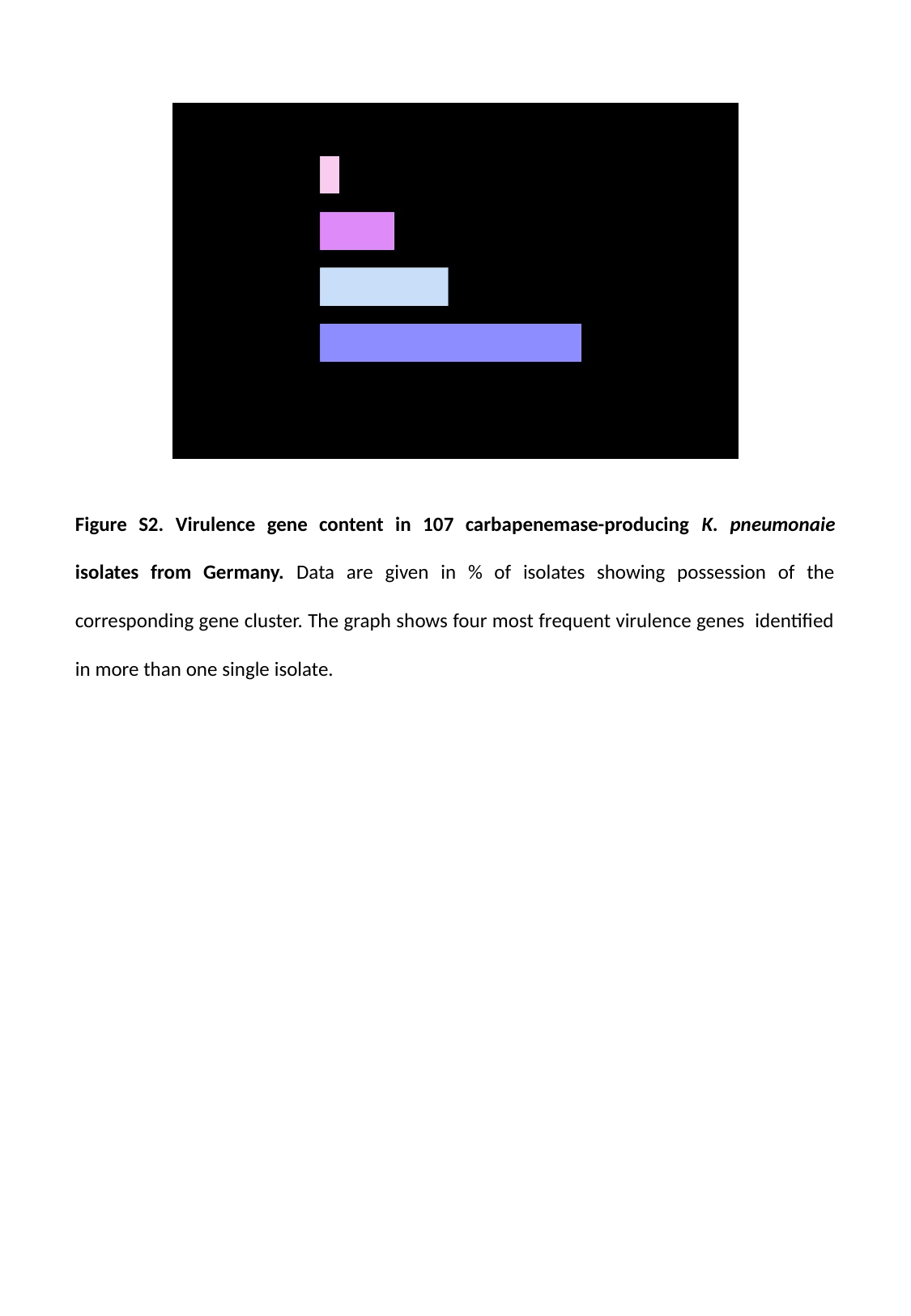

Figure S2. Virulence gene content in 107 carbapenemase-producing K. pneumonaie isolates from Germany. Data are given in % of isolates showing possession of the corresponding gene cluster. The graph shows four most frequent virulence genes identified in more than one single isolate.

## Slide 3
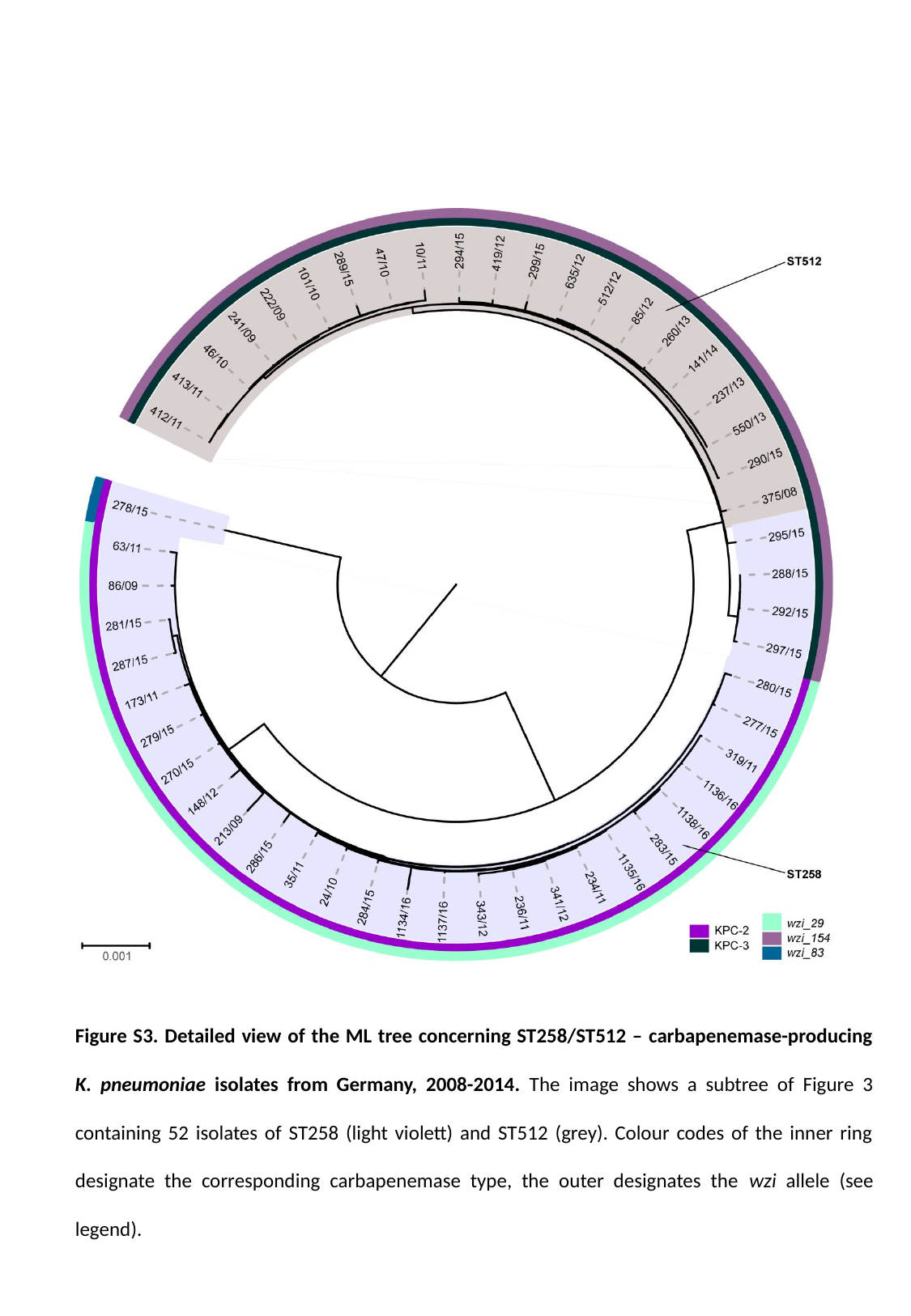

Figure S3. Detailed view of the ML tree concerning ST258/ST512 – carbapenemase-producing K. pneumoniae isolates from Germany, 2008-2014. The image shows a subtree of Figure 3 containing 52 isolates of ST258 (light violett) and ST512 (grey). Colour codes of the inner ring designate the corresponding carbapenemase type, the outer designates the wzi allele (see legend).

## Slide 4
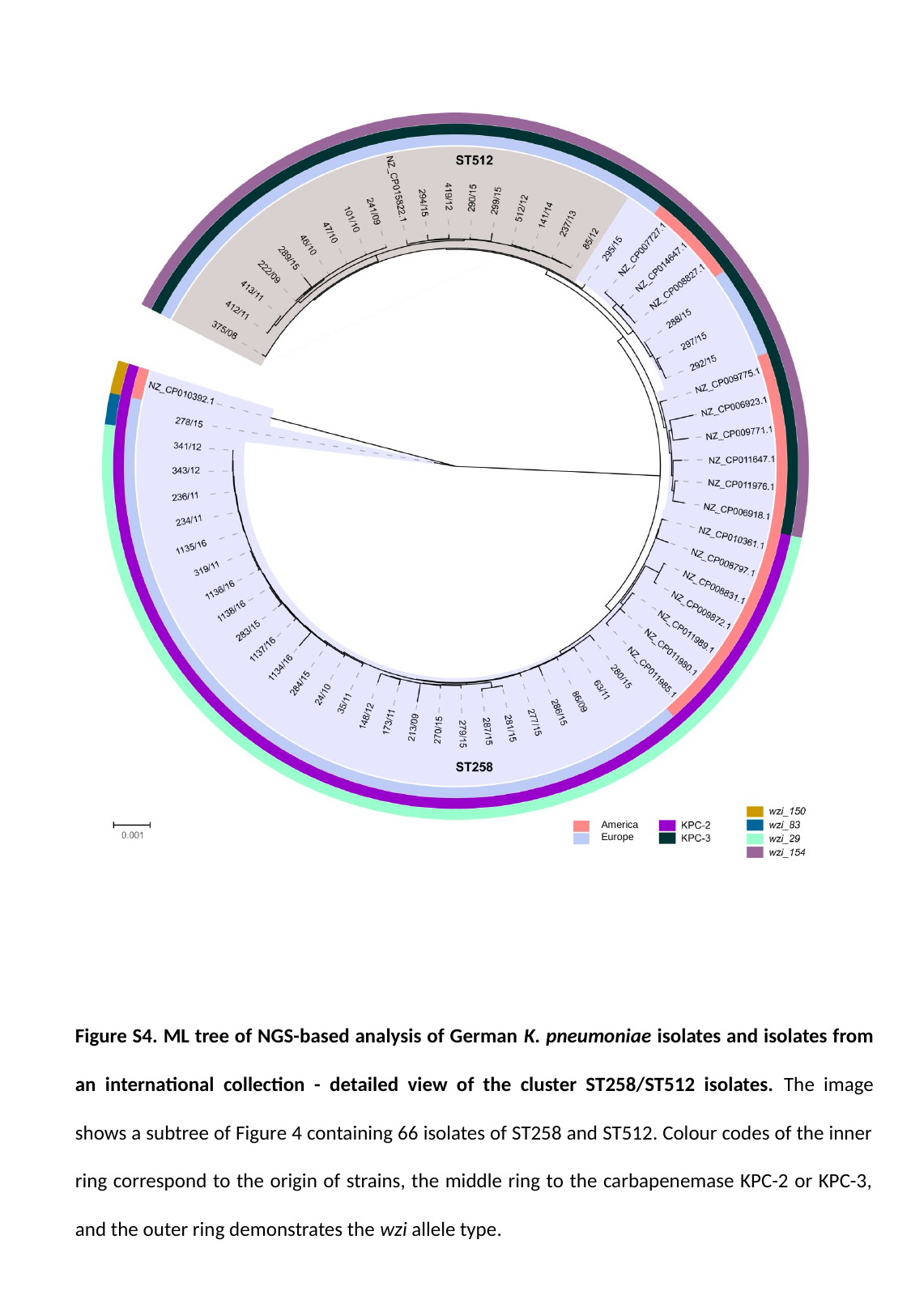

America
Europe
Figure S4. ML tree of NGS-based analysis of German K. pneumoniae isolates and isolates from an international collection - detailed view of the cluster ST258/ST512 isolates. The image shows a subtree of Figure 4 containing 66 isolates of ST258 and ST512. Colour codes of the inner ring correspond to the origin of strains, the middle ring to the carbapenemase KPC-2 or KPC-3, and the outer ring demonstrates the wzi allele type.
