# Supplementary material for: Genome-based analysis of Carbapenemase-producing Klebsiella pneumoniae isolates from German hospital patients, 2008-2014
Source: Antimicrob Resist Infect Control. 2018 May 2;7:62. doi: 10.1186/s13756-018-0352-y (PMC5930415; doi:10.1186/s13756-018-0352-y)
Supplement: Supplementary file 3 — Table S2. Names and sequences of used primers for MLST and PCR amplification of virulence genes and β-lactamase genes. Table S3. Identified virulence genes in K. pneumoniae isolate no. 316/15 (ST23, OXA-48). Table S4. Identified wzi alleles from NGS data of 107 carbapenemase-producing K. pneumoniae from Germany. (DOCX 35 kb) [file 13756_2018_352_MOESM3_ESM.docx]

**Table S2. Names and sequences of used primers for MLST and PCR amplification of virulence genes and β-lactamase genes.**

| **Primer name** | **Sequence (5‘-3‘)** | **PCR conditions** | **Reference** |
| --- | --- | --- | --- |
| wzi_for2 | GTGCCGCGAGCGCTTTCTATCTTGGTATTCC | 94 °C 2 min, (94 °C 30 s, 55 °C 40 s, 72 °C 30 s) ×30, 72 °C, 5 min | (Brisse et al. 2013) |
| wzi_rev | GAGAGCCACTGGTTCCAGAAYTTSACCGC | 94 °C 2 min, (94 °C 30 s, 55 °C 40 s, 72 °C 30 s) ×30, 72 °C, 5 min | (Brisse et al. 2013) |
| wzi-new-F | CTGCATCTACCAGTGTTCCCCTTGTCG | 94 °C 2 min, (94 °C 30 s, 55 °C 40 s, 72 °C 30 s) ×30, 72 °C, 5 min | This paper. |
| SHV rev (SHVu) | GGTTAGCGTTGCCAGTGCT | 95 °C 5 min, (95 °C 30 s, 55 °C 30 s, 72 °C 1 min)x30, 72 °C, 5 °min | (Gröbner et al. 2009) |
| SHV-FWD-neu (SHVu) | GCAAAACGCCGGGTTATTC | 95 °C 5 min, (95 °C 30 s, 55 °C 30 s, 72 °C 1 min) ×30, 72 °C, 5 °min | (Gröbner et al. 2009) |
| OXA-1-ges F | tatctacagcagcgccagtg | 95 °C 5 min, (95 °C 30 s, 60 °C 30 s, 72 °C 1 min) ×30, 72 °C, 5 min | (Pfeifer et al. 2011) |
| OXA-1-ges R | taaattcgaccccaagtttcc | 95 °C 5 min, (95 °C 30 s, 60 °C 30 s, 72 °C 1 min) ×30, 72 °C, 5 min | (Pfeifer et al. 2011) |
| infb-F | GTTTTCCCAGTCACGACGTTGTA**CTCGCTGCTGGACTATATTCG** | 95 °C 2 min, (95 °C 15 s, 50 °C 30 s, 72 °C 1 min) ×35, 72 °C, 4 min | ^1)^ |
| infb-R | TTGTGAGCGGATAACAATTTC**CGCTTTCAGCTCAAGAACTTC** | 95 °C 2 min, (95 °C 15 s, 50 °C 30 s, 72 °C 1 min) ×35, 72 °C, 4 min | ^1)^ |
| pgi-F | GTTTTCCCAGTCACGACGTTGTA**GAGAAAAACCTGCCTGTACTGCTGGC** | 95 °C 2 min, (95 °C 15 s, 50 °C 30 s, 72 °C 1 min) ×35, 72 °C, 4 min | ^1)^ |
| pgi-R | TTGTGAGCGGATAACAATTTC**CGCGCCACGCTTTATAGCGGTTAAT** | 95 °C 2 min, (95 °C 15 s, 50 °C 30 s, 72 °C 1 min) ×35, 72 °C, 4 min | ^1)^ |
| phoe-F | GTTTTCCCAGTCACGACGTTGTA**ACCTACCGCAACACCGACTTCTTCGG** | 95 °C 2 min, (95 °C 15 s, 50 °C 30 s, 72 °C 1 min) ×35, 72 °C, 4 min | ^1)^ |
| phoe-R | TTGTGAGCGGATAACAATTTC**TGATCAGAACTGGTAGGTGAT** | 95 °C 2 min, (95 °C 15 s, 50 °C 30 s, 72 °C 1 min) ×35, 72 °C, 4 min | ^1)^ |
| gapa-F | GTTTTCCCAGTCACGACGTTGTA**TGAAATATGACTCCACTCACGG** | 95 °C 2 min, (95 °C 15 s, 50 °C 30 s, 72 °C 1 min) ×35, 72 °C, 4 min | ^1)^ |
| gapa-R | TTGTGAGCGGATAACAATTTC**CTTCAGAAGCGGCTTTGATGGCTT** | 95 °C 2 min, (95 °C 15 s, 50 °C 30 s, 72 °C 1 min) ×35, 72 °C, 4 min | ^1)^ |
| mdh-F | GTTTTCCCAGTCACGACGTTGTA**CCCAACTCGCTTCAGGTTCAG** | 95 °C 2 min, (95 °C 15 s, 50 °C 30 s, 72 °C 1 min) ×35, 72 °C, 4 min | ^1)^ |
| mdh-R | TTGTGAGCGGATAACAATTTC**CCGTTTTTCCCCAGCAGCAG** | 95 °C 2 min, (95 °C 15 s, 50 °C 30 s, 72 °C 1 min) ×35, 72 °C, 4 min | ^1)^ |
| tonb1-F | gatttacctcgccgctttc | 95 °C 2 min, (95 °C 15 s, 60 °C 30 s, 72 °C 1 min) ×35, 72 °C, 4 min | This paper |
| tonb1-R | cggtcttccctgctgataac | 95 °C 2 min, (95 °C 15 s, 60 °C 30 s, 72 °C 1 min) ×35, 72 °C, 4 min | This paper |
| MLST-KP-F | GTTTTCCCAGTCACGACGTTGTA | Sequenzierung, T_A_ 50 °C | ^1)^ |
| MLST-KP-R | TTGTGAGCGGATAACAATTTC | Sequenzierung, T_A_ 50 °C | ^1)^ |
| rpoB-F | GTTTTCCCAGTCACGACGTTGTA**GGCGAAATGGCWGAGAACCA** | 95 °C 2 min, (95 °C 15 s, 50 °C 30 s, 72 °C 1 min) ×35, 72 °C, 4 min | ^1)^ |
| rpoB-R | TTGTGAGCGGATAACAATTTC**GAGTCTTCGAAGTTGTAACC** | 95 °C 2 min, (95 °C 15 s, 50 °C 30 s, 72 °C 1 min) ×35, 72 °C, 4 min | ^1)^ |

^1)^ <http://bigsdb.pasteur.fr/klebsiella/primers_used.html>

**References**

[Brisse S](https://www.ncbi.nlm.nih.gov/pubmed/?term=Brisse%20S%5BAuthor%5D&cauthor=true&cauthor_uid=24088853), [Passet V](https://www.ncbi.nlm.nih.gov/pubmed/?term=Passet%20V%5BAuthor%5D&cauthor=true&cauthor_uid=24088853), [Haugaard AB](https://www.ncbi.nlm.nih.gov/pubmed/?term=Haugaard%20AB%5BAuthor%5D&cauthor=true&cauthor_uid=24088853), [Babosan A](https://www.ncbi.nlm.nih.gov/pubmed/?term=Babosan%20A%5BAuthor%5D&cauthor=true&cauthor_uid=24088853), [Kassis-Chikhani N](https://www.ncbi.nlm.nih.gov/pubmed/?term=Kassis-Chikhani%20N%5BAuthor%5D&cauthor=true&cauthor_uid=24088853), [Struve C](https://www.ncbi.nlm.nih.gov/pubmed/?term=Struve%20C%5BAuthor%5D&cauthor=true&cauthor_uid=24088853), [Decré D](https://www.ncbi.nlm.nih.gov/pubmed/?term=Decr%C3%A9%20D%5BAuthor%5D&cauthor=true&cauthor_uid=24088853). *wzi* Gene sequencing, a rapid method for determination of capsular type for *Klebsiella* strains. [J Clin Microbiol.](https://www.ncbi.nlm.nih.gov/pubmed/?term=brisse+wzi+2013) 2013 Dec;51(12):4073-8.

Gröbner S, Linke D, Schütz W, Fladerer C, Madlung J, Autenrieth IB, Witte W, Pfeifer Y. [*Emergence of carbapenem-non-susceptible extended-spectrum beta-lactamase-producing Klebsiella pneumoniae* isolates at the university hospital of Tübingen, Germany.](https://www.ncbi.nlm.nih.gov/pubmed/19502377) J Med Microbiol. 2009; 58:912-22.

Pfeifer Y, Wilharm G, Zander E, Wichelhaus TA, Göttig S, Hunfeld KP, Seifert H, Witte W, Higgins PG. [Molecular characterization of blaNDM-1 in an *Acinetobacter baumannii* strain isolated in Germany in 2007.](https://www.ncbi.nlm.nih.gov/pubmed/21693460) J Antimicrob Chemother. 2011; 66:1998-2001.

**Table S3. Identified virulence genes in *K. pneumoniae* isolate no. 316/15 (ST23, OXA-48).**

Possession of virulence genes is inferred from NGS data using the virulence gene database of the Pasteur Institute by denominating the factor/cluster and the corresponding genomic loci (<http://bigsdb.pasteur.fr/perl/bigsdb/bigsdb.pl?db=pubmlst_klebsiella_seqdef_public&page=downloadAlleles&scheme_id=4&render=1>)

| **Virulence factor** | **Locus allele** |
| --- | --- |
| **mrk** | *mrkA*_1, *mrkB*_1, *mrkC*_1, *mrkD*_1, *mrkF*_1, *mrkH*_1, *mrkI*_2, *mrkJ*_1, |
| **Yersiniabactin** | *ybtA*_2, *ybtE*_2, *ybtP*_2, *ybtQ*_2, *ybtS*_ 2, *ybtT*_neu, *ybtU*_2, *ybtX*_2, *irp1*_6, *irp2*_36, *fyuA*_2 |
| **Allantionase** | *allA*_1, *allB*_1, *allC*_2, *allD*_1, *allR*_1, *allS*_1, *ylbE*_1, *ylbF*_1, *gcl*_2, *fdrA*_1, *arcC*_1, *ybbW*_1, *ybbY*_1, *hyi*_1, *glxK*_1, *glxR*_1 |
| **Colibactin** | *clbA*_2, *clbB*_2, *clbC*_2, *clbD*_2, *clbE*_2, *clbF*_2, *clbG*_2, *clbH*_3, *clbI*_2, *clbJ*_neu, *clbK*_5, *clbL*_2, *clbM*_2, *clbN*_2, *clbO*_2, *clbP*_2, *clbQ*_2, *clbR*_2 |
| **Salmochelin** | *iroB*_1, *iroC*_4, *iroD*_1, i*roN*_1 |
| **Aerobactin** | *iutA*_1, *iucA*_neu, *iucB*_1, *iucC*_1, *iucD*_1 |
| **kfu** | *kfuA*_1, *kfuB*_1, *kfuC*_1 |
| **Microcin** | *mceA*_1, *mceB*_1, *mceC*_1, *mceD*_2, *mceE*_1, *mceG*_2, *mceH*_1, *mceI*_2, *mceJ*_2 |
| **rmpA** | *rmpA*_2 |
| **rmpA2** | *rmpA2*_8 (*Frameshift*) |

**Table S4. Identified *wzi* alleles from NGS data of 107 carbapenemase-producing *K. pneumoniae* from Germany.**

The table shows the number of *wzi* alleles per MLST types. The corresponding capsule type was inferred from the *wzi* allele according to Brisse et al. 2013. More than one capsule type per *wzi* type separated by a comma refers to the fact that corresponding strains reacted with several antisera. Two capsule types combined by a plus sign refer to corresponding isolates which reacted with both sera.

| ***K. pneumoniae***  **MLST type** | **Number** | ***wzi* allele** | **Associated capsule type**  (Brisse et al. 2013) |
| --- | --- | --- | --- |
| **ST11** | n = 1 | *wzi*_177 |  |
| **ST15** | n = 4 | *wzi*_24 (n = 2) | K24 |
|  |  | *wzi*_19 (n = 1) | K19 |
|  |  | *wzi*_552 (n = 1) |  |
| **ST16** | n = 2 | *wzi*_50 | K15, K17, K50, K51, K52 |
| **ST17** | n = 1 | *wzi*_555 |  |
| **ST23** | n = 1 | *wzi*_1 | K1 |
| **ST37** | n = 1 | *wzi*_96 | K38 |
| **ST48** | n = 1 | *wzi*_167 |  |
| **ST101** | n = 16 | *wzi*_137 | K17 |
| **ST147** | n = 13 | *wzi*_64 (n = 11) | K64 + K14 |
|  |  | *wzi*_12 (n = 1) | K12, K29 |
|  |  | unknown (n = 1) |  |
| **ST258** | n = 31 | *wzi*_29 (n = 26) | K41 |
|  |  | *wzi*_154 (n = 4) |  |
|  |  | *wzi*_83 (n = 1) | K23 |
| **ST307** | n = 1 | *wzi*_173 |  |
| **ST340** | n = 2 | *wzi*_50 | K15, K17, K50, K51, K52 |
| **ST347** | n = 1 | *wzi*_553 |  |
| **ST391** | n = 1 | *wzi*_554 |  |
| **ST395** | n = 3 | *wzi*_2 | K2 |
| **ST512** | n = 21 | *wzi*_154 |  |
| **ST629** | n = 1 | *wzi*_100 | K10 |
| **ST784** | n = 1 | *wzi*_118 |  |
| **ST906** | n = 1 | *wzi*_508 |  |
| **ST1399** | n = 3 | *wzi*_43 | K43 |
| **ST2254** | n = 1 | *wzi*_85 | K30 |

**References**

[Brisse S](https://www.ncbi.nlm.nih.gov/pubmed/?term=Brisse%20S%5BAuthor%5D&cauthor=true&cauthor_uid=24088853), [Passet V](https://www.ncbi.nlm.nih.gov/pubmed/?term=Passet%20V%5BAuthor%5D&cauthor=true&cauthor_uid=24088853), [Haugaard AB](https://www.ncbi.nlm.nih.gov/pubmed/?term=Haugaard%20AB%5BAuthor%5D&cauthor=true&cauthor_uid=24088853), [Babosan A](https://www.ncbi.nlm.nih.gov/pubmed/?term=Babosan%20A%5BAuthor%5D&cauthor=true&cauthor_uid=24088853), [Kassis-Chikhani N](https://www.ncbi.nlm.nih.gov/pubmed/?term=Kassis-Chikhani%20N%5BAuthor%5D&cauthor=true&cauthor_uid=24088853), [Struve C](https://www.ncbi.nlm.nih.gov/pubmed/?term=Struve%20C%5BAuthor%5D&cauthor=true&cauthor_uid=24088853), [Decré D](https://www.ncbi.nlm.nih.gov/pubmed/?term=Decr%C3%A9%20D%5BAuthor%5D&cauthor=true&cauthor_uid=24088853). *wzi* Gene sequencing, a rapid method for determination of capsular type for *Klebsiella* strains. [J Clin Microbiol.](https://www.ncbi.nlm.nih.gov/pubmed/?term=brisse+wzi+2013) 2013 Dec;51(12):4073-8.
